# Experiences of being a young male Sami reindeer herder: a qualitative study in perspective of mental health

**DOI:** 10.3402/ijch.v72i0.20926

**Published:** 2013-07-08

**Authors:** Niclas Kaiser, Terje Ruong, Ellinor Salander Renberg

**Affiliations:** 1 Department of Psychology, Umeå University, Umeå, Sweden; 2 Department of Clinical Sciences, Division of Psychiatry, Umeå University, Umeå, Sweden

**Keywords:** Sami, mental health, reindeer herder, males, gender, experiences, qualitative content analysis, organizational culture

## Abstract

**Objectives:**

To explore experiences of what it is to be a young male Sami reindeer herder in Sweden, a group with previously known stigma and specific health issues, and to understand experiences in perspective of mental health.

**Methods:**

A qualitative content analysis was employed. Data were collected by in-depth interviews with 15 strategically selected reindeer herders aged 18–35 years old.

**Results:**

The analysis resulted in 5 sub-themes: (a) being “inside” or “outside” is a question of identity; (b) a paradox between being free/bound; (c) an experience of various threats and a feeling of powerlessness; (d) specific norms for how a “real” reindeer herder should be; and (e) the different impacts and meanings of relations. The overarching theme is summarized thus: being a young reindeer herder means so many (impossible) dreams and conditions. Overall, the experience of the informants was that being a reindeer herder is a privileged position that also implies many impossibilities and unjust adversities they have no control over, and that there is nothing they can do but “bite the bullet or be a failure.”

**Conclusions:**

Knowledge about this group's experiences can be used to understand difficulties faced by young reindeer herders and its consequences regarding mental health problems. This also implies a need for a broader perspective when discussing future interventions aimed at preventing mental health problems in this group.

Swedish reindeer herders are part of the Sami indigenous population, who live mainly in the region known as Sápmi (northern Scandinavia and the Kola Peninsula). Estimates of the size of the Sami population differ depending on what criteria are used, but approximately 75,000–100,000 in total, mostly living in Norway, with about 20,000 in Sweden. In general, and in international comparison, the health situation of the Swedish Sami population is good, despite reports of discrimination (1). There are some 2,000 individuals over the age of 18 subsisting totally or partly on reindeer herding in Sweden, and they constitute 5–10% of the Swedish Sami population. This group has been described as a Sami core group and the most important cultural bearer of Sami identity (2,3). Regarding health, earlier studies have shown that reindeer herding is one of the most dangerous occupations in Sweden (4), and further research has shown that the work situation of reindeer herders is characterized by a difficult financial situation, high demands, little control, a low level of social support and little hope for the future (5,6). A recent cross-sectional study on mental health among Swedish reindeer herders concluded that the symptom levels of depression and anxiety were higher among reindeer herders than in geographically matched reference populations (7). Only small differences were found regarding use of alcohol (8). As Indigenous peoples are generally vulnerable to suicidal expressions, and young indigenous males in particular (9), the most alarming result is perhaps the higher prevalence of various types of suicidal problems and exposure to suicidal behaviour in the Swedish Sami reindeer herding population (10).

During 18th and 19th century colonization of Sápmi, the Swedish government had the idea to protect the Sami culture from the impact of the developing modern civilization, as the Sami lifestyle was considered to be hard and primitive. The Sami were at the same time considered as legally incompetent. The Sami were also wrongly considered to be solely nomadic and should therefore not be in need of owning land. The rights of the Sami people in relation to the non-Sami population were stipulated as the right to use land for reindeer herding, fishing and hunting (11). As these rights applied only to those who, at the time, could subsist on reindeer herding, the situation split the Sami population into 2 groups, reindeer herders and non-reindeer herders. In legal matters, only reindeer herders were considered as Sami. The non-reindeer herding Sami were thereby not considered as “problematic,” as they were not “real Sami,” and still not “real Swedes” (12). At the same time, the right to use the same land was given to both Swedish landowners and Sami reindeer herders. Partly as a result of this legislation, reindeer herders now have a specific position as bearers of the most important symbol of Sami culture (2), reindeer herding itself. This situation, which still exists after 130 years, is a source of conflict on several levels. To administer the rights of the Sami reindeer herding population, the Swedish government created the organizational structure of Sami villages (*samebyar*) and only members of such villages were allowed to use land that had traditionally been used by all Sami people. To this day, only Sami are allowed to become members of these villages, and the board of the Sami village decides who is to become a member and thereby who is allowed to be a Sami reindeer herder.

Reindeer herding has gone through tremendous change over the past 30 years and is now a highly motorized business. Generally the reindeer are herded on grazing lands in mountain areas during summer and in forests and along the coast during winter. These circumstances differ depending on whether the traditionally used area is located in the south or north of Sápmi and on the type of Sami village (i.e. forest or mountain).

Describing the cultural development in terms of acculturation (13) shows us a situation in which the Sami community has gone through several different processes with specific health consequences. While much of the Sami population has been assimilated into the main Swedish society, the male reindeer herders are described as having been separated from it. Reindeer herding women on the other hand have a position of “one foot in each culture” as they often are highly educated (7) and have a pronounced responsibility for earning a monthly income, which makes them more integrated than the men (1) and thereby more in contact with, for example, health services. There are recent reports on suicidal expressions among young Sami (14), discrimination and ethnic-related ill treatment, especially highlighting young reindeer-herders experiences of ethnic related ill treatment (15), concluding with the need for further research in the aim to aid the majority culture's understanding and to assist in producing a culture-adapted health care.

The present study focuses on the situation of young male reindeer herders as motivated by their core position in Sami society and the special attention that has been devoted to health risk exposure among young indigenous persons (16–18) and young indigenous men in particular as they generally are less prone to use health care services, are less educated and have higher proportions of alcohol abuse and mental problems (19). This study thereby covers the need to deepen our understanding of what it is to be a young male reindeer herder, as presented through their own experiences.

## Methods

The study had an explorative design, applying an inductive approach through qualitative content analysis (20). Data were collected through 15 qualitative interviews lasting 60–90 minutes. To get a heterogeneous group of interviewees and to increase possibilities for variation and richness in the interview data, the interviewees were selected according to geographical group (South Sami, Lule Sami and North Sami) and type of reindeer herding village (forest or mountain) within the age group of 18–35 years. The informants were contacted by telephone and also received information about the research project by e-mail. As the study's remit did not include differences regarding areas of living, the groups were collapsed into one. Out of respect for confidentiality, information about the informants is not presented in table form. The interviews, which were conducted with each informant at the location of their choice (in their home or in rooms facilitated by the research team), were carried out by one of the 2 researchers: NK, clinical psychologist and non-Sami, or TR, reindeer-herding Sami, writing a master thesis within the psychologist program. The interviews were conducted during October 2009, following a semi-structured interview guide. They were then audio-recorded and transcribed verbatim. The interviews began with open questions about personal experiences of what makes the informant feel good or bad, and what he does in these situations. These questions covered symptoms of depression and anxiety, as well as coping strategies, without using leading questions. The interviewer encouraged the informants to speak freely about their thoughts and feelings regarding different aspects of the themes probed by the interviewer, such as “thoughts about suicide,” “use of alcohol” and “significance of gender.” After the first interview was conducted, minor adjustments were made in the interview guide.

The language used for the all interviews was Swedish. All interviewees said that they would not express themselves more nuanced or richly in Sami, and no interpreter was needed. In fact many informants stated that it would have been awkward for them to speak Sami, as their everyday language was Swedish. The results part of this article were first written in Swedish in order not to miss important aspects in the material. While the original interviews were in Swedish, some of the nuances in the presented interview-extracts have been lost in the translation process, even though it was reviewed by a native-English professional proof reader.

The study was approved by the regional research ethics committee in Umeå, Sweden, and the Helsinki Declaration has been respected (21), focusing on informed consent, confidentiality and care for the participants.

### Data analysis

The data were analysed using qualitative content analysis according to Graneheim and Lundman (22). The aim of such analysis is to produce a detailed, systematic recording of the themes and issues addressed in the data. The main focus is on describing, summarizing and interpreting the manifest content, thereby enhancing themes covering latent content in the material. An inductive design was used; that is, themes and their contents were not derived from any specific theoretical perspective. For instance, the participants elaborations on the specific questions probed in the interviews were not treated as thematically separated in the analysis. This was conducted through a stepwise process of abstracting meanings without losing contact with the original data in the text. The transcribed interviews were managed in the QRS NVIVO software program (NVivo 8), allowing the research team to continuously follow the link between raw text and the main theme through codes, categories and sub-themes. The coding was mainly conducted by TR, but the development of categories and themes was carried out in full collaboration.

The analysis was made through different phases, as follows:

- A naïve reading and rereading of the texts, giving a sense of the whole. Texts not concerning the issues of the study were separated out and excluded.
- Nine interviews were selected as heterogeneous enough to provide a widespread variety of information. This selection was made using criteria as “information-richness” and “uniqueness” and with strong intercoder reliability. The remaining 6 interviews did not therefore go through the following procedure, but played an important role in giving a sense of the whole.
- The text was divided into meaning units, that is, sentences containing aspects related to the study, which in turn were condensed, that is, shortening the meaning while still preserving the core.
- The condensed meanings were abstracted and labelled with codes. The codes were continuously compared for similarities and differences through a process of reflection and discussion among the researchers. This intercoder reliability was strengthened by first coding separately and then comparing codes and understandings of codes. This resulted in agreements about how to sort the codes into categories, and later on, how to sort the categories into sub-themes. Finally, five sub-themes emerged that seemed to serve as relevant headings to unify the categories, with one main theme to unify the sub-themes. Examples of the analysis, with raw text, meaning unit, condensed meaning unit, code, category, sub-theme and theme, are presented in Table I.

**Table I:** Examples of raw text, meaning units, condensed meaning units, codes, categories and themes

Raw text	Meaning units	Condensed meaning units	Code	Category	Sub-theme	
No, I think it's almost impossible, and I think that leaving and taking another job would feel like a defeat, you didn't make it living as reindeer herder, he failed. I think it's a big defeat thing, but then there's a big, I mean you're born and raised into it. I mean, you're raised into that life, you want to live it. And then it doesn't work and then it can become a dark tunnel that doesn't get light again, which is probably quite hard.	Almost impossible to quit reindeer herding, that would be like a defeat. A failure not to make it as reindeer herder, then you have been defeated. Those who are reindeer herders are born into it. If you are raised as reindeer herder you want to live that life. If it doesn't work out to live as reindeer herder it can be like a dark tunnel that doesn't get light again.	Hard to stop reindeer herding, that would be a defeat. If you can't live as a reindeer herder you have failed. You are born into reindeer herding. If you are raised as a reindeer herder you want to live that life. If it doesn't work out to live as reindeer herder it can be like a dark tunnel that doesn't get light again.	A defeat to give up being a reindeer herder A failure if you give up reindeer herding. You are born into reindeer herding. If you are raised as a reindeer herder you want to live that life. It will be dark and difficult if you can't continue as a reindeer herder.	A failure if you give up being a reindeer herder As a reindeer herder, you should have special traits. A failure if you quit reindeer herding	There are specific norms for how a good reindeer herder should be	Being young reindeer herder means so many (impossible) dreams and conditions

## Results

The main theme and sub-themes are presented in Table II. In the following, each sub-theme is described. The categories are integrated into the text and illuminated with relevant quotations. Due to the character of the main theme, it is presented last.

**Table II:** Main theme and sub-themes concerning experiences of being a young male reindeer herding Sami in Sweden

Being a young reindeer herder means so many (impossible) dreams and conditions
Being “inside” or “outside” is a matter of identity
There is a paradox between being free/bound
An experience of various threats and a feeling of powerlessness
Specific norms for how a “real” reindeer herder should be
The different impacts and meanings of relations

### Being “inside” or “outside” is a matter of identity

Reindeer herding is described more as a lifestyle than a profession, and as something that constantly occupies one's life and world. Being a reindeer herder is an identity and something you are born into, but requires great interest on the part of the person who is or is to become a reindeer herder. On the other hand, if you were born a reindeer herder, you probably have this interest from birth. This means that you have also received the “proper” norms and values from those around you in your immediate family, extended family and Sami village. As one informant put it:

My world is reindeer herding, seven days a week, my life, my dreams, my family circumstances; everything gets like that because it occupies so much … of everything surrounding it … Which I like, but … it's immense, it's everything you do and also everything you're associated with.

Reindeer herding, which is understood as the centre of Sami culture, is said to give high status in the Sami world. Overall, reindeer herding makes herders stand out from the others, and they have a culture of their own that they are very proud of, as witnessed by these statements from 2 informants:

You get the vibes [that say] that you are higher in rank in the Sami world in general if you are a reindeer herder, and if you have a lot of reindeer, you are of an even higher rank.

You have an identity … That's what it's like, you're not just an average Swede.

It is important to stick to the culture and maintain borders towards other groups, like defending legal rights for reindeer herders. These following quotes reflect this:

If you take that away [reindeer herding rights] then there's nothing that differs between a regular Swede and a reindeer herder … and I think the Sami and reindeer herders in general should be allowed to continue to have this pride.

Not everyone has the opportunity to live like this. So I think those who get the chance should think both twice and three times before they totally reject it.

Numbers of reindeer herders are declining, and the community needs young people to secure regrowth and make sure family names in reindeer herding are passed on. The decrease is both good (gives your children space and increases your own possibilities because of less competition for grazing land) and bad (more work). Therefore, it is not a given that you let new reindeer herders into the Sami village, which leads to a situation where herders cannot give up reindeer herding, as it would be difficult or impossible to return to the profession once they have left. As one informant expressed it:

Now we are very few, and we have a lot of grazing land and I have all the odds on my side … And that's the most important thing, that we don't get more reindeer herders … I can develop my herd to the max.

Because reindeer herding is a matter of identity, who are you if you give it up? You have nothing left, and you would have disappointed your relatives, not to mention your own emotional ties to the grazing land. What would happen if you were forced to leave? As one informant asked himself: “Who am I if I stop working with reindeer? I would have nothing of myself left.”

### Paradox of being free yet bound

There are contradictory aspects of freedom in relation to choosing reindeer herding as an occupation and regarding the character of reindeer herding per se, as well as specific social and gendered consequences. The choice is claimed to be made freely, but at the same time with a feeling of being bound, as it is so important to carry on the family line of reindeer herders, you are more or less forced to be a reindeer herder if you are given the opportunity. Three informants expressed ideas surrounding this conflict thus:

I feel no pressure that I have to, on the contrary, it's my choice, and I would very much like my children to be able to choose [to be reindeer herders] as well.

My father would probably be … he has never forced me or asked me to make an active choice, but I think he would be very disappointed [if I gave up reindeer herding], yeah, I think so.

It would make me very upset … to tell the truth [if his children did not want to continue reindeer herding]). It would be … well, hopeless.

Life as a reindeer herder is described as free, but also totally bound. Bound to the reindeer which always come foremost, and the reindeer's needs are unpredictable. Because of this, and the very long hours away from home, there are difficulties in making plans or meet with family and friends on a regular basis, and this is expressed as a feeling of having to sacrifice social and family life. This is also offered as the reason why women cannot be reindeer herders, at least not after they have given birth. But you cannot afford to support a family of your own on reindeer herding alone, so female spouses have to have a job with a steady income. It is important that a potential girlfriend understands the circumstances of reindeer herding. It is preferable that they come from reindeer herding families and are of the “old school” who know that their men cannot stay at home with the children on paternity leave and that it is their task to be the primary caretaker of the home and children. Finding such a girlfriend is described as getting more and more difficult. Some quotes that reflect this are:

At the same time, it's so expensive, [so] if you have a family and children there isn't enough money [in reindeer herding], to be frank, one of you has to have another job.

Above all it's up to the girl, I mean how great her understanding is and well, they're home alone with the children, that's just the way it is … at the same time there are many who have that new attitude towards gender equality, there are many who have a problem with that.

### Threats and a feeling of powerlessness

Many reindeer herders experience various kinds of threats to their way of living, for example, from the surrounding society in the form of land intrusions from mining companies or from nature in the form of predators that disturb, disperse or kill among the herd. They express this thus:

What has affected me negatively the last two, at least two, three years is this growing population of predators. This powerlessness, the powerlessness you felt at the time, also the feeling, regarding authorities, that you haven't been listened to.

There are other threatening clouds in the form of a lack of being able to foresee the financial situation or the weather conditions that determine financial outcome and workload. As one reindeer herder put it:

[Income] is everything in reindeer herding. There are so many things that depend on money to make it possible to subsist on reindeer herding. It's so easy to be knocked over by a bad year of calving, or high pressure from predators or poor grazing conditions, and it can run up to hundreds of thousands of crowns … that can beat anyone down … you have to think so long-term and you know so very little about what's going to happen.

In addition, it appears to be very difficult to get a rest. Another problem is that the reindeer are exposed to dangers when they are in populated areas.

There are also conflicts with other Sami villages about the right to use specific grazing lands, sometimes leading to threats, violence and police reports, making young reindeer herders feel uncomfortable when driving out to look after the herd:

You almost get scared when you hear another snowmobile in the forest because, just because there's been a quarrel about who, well, who lost their reindeer where and who came there on purpose and who has the right to be there and graze the land … I mean, it's no fun to drive out to the forest in the morning if you're afraid, yeah, you get afraid to meet people, you're afraid you'll have to get into a quarrel, that's been really tough.

The men experience media descriptions of reindeer herders and reindeer herding as both offensive and upsetting. They experience prejudiced beliefs about reindeer herders as destroyers of land and dependent on benefits, sometimes questioning the traditional Sami right to let reindeer graze the land at all. Taken together, the young men in the study describe a feeling of lack of understanding from almost everyone outside the reindeer herding community, and it is their experience that there is nothing they can do to change the situation, except among their nearest non-Sami acquaintances. In conversations about feeling persecuted by governmental authorities, they express a lack of understanding on the part of the Swedish Government and ask themselves what the future of reindeer herding will be. All of these misunderstandings and lack of knowledge on the part of others lead to isolation within the group, which is described as difficult, as you often feel you are not a part of the general population.

There are also problems within the Sami villages. Anger sometimes wells up when young reindeer herders have to sacrifice money, time and social life while others in the Sami village are “loafers” at their expense. In those cases, the men in our study described reindeer herding legislation as obsolete, as reindeer herding is a right you cannot be dismissed from. There are other conflicts as well, such as young vs. old, competition on different levels and heritage.

### Living with specific norms for how a “real” reindeer herder should be

The young reindeer herders perceived norms from especially within their own group. They felt you “had” to have an interest in reindeer herding, and that is what makes you continue. It is never the surrounding circumstances that would force you to leave, only your own lack of “interest,” and interest comes with upbringing and being of true Sami “grit.” So if you leave the occupation, you simply were not the right person for it. As one Sami expressed it: “Reindeer herding is such that if you aren't interested, nothing will come of it, everything will just be a total mess.” There is a macho culture involved, and you should be outdoors the longest, come back to the hut the latest, withstand the worst weather and stay at the furthest edge of the herd. If you experience trouble, you endure and work harder. You are not supposed to complain or show any sign of weakness.

It's a macho culture, it just is, you're supposed to be tough, not show when you're feeling bad and not show that your body isn't up to it, because, yeah, then you're weak.

[In times of trouble] I clench my teeth, spit into my hands and work harder, pretty much. That's all you can do. I mean, I'm not the kind of person who goes around waiting for other people to solve things, I'm not.

The young men talk about the fact that there are many things they should and should not talk about. They do not talk about problems if they are not of a technical character. If they are depressed and brooding, they keep it to themselves and do not seek help from professionals. As 2 informants said:

I'm a pretty “closed” person who solves most things on my own and who doesn't like to talk about problems.

Going so far as to go and seek help, you know, talk to a psychologist, that's a very big step. It's a huge step … you're not supposed to show that you have any problems. No one, you don't want anyone to know you have any problems at all, whether they're economical or …, you should keep a facade outwards.

The informants feel they are supposed to know everything without asking. There is an assumption that others will understand the signals they show, without talking. Both acknowledgement and criticism are given through “detours.” This leads to problems when they want to criticize but cannot, as evidenced by these quotes:

There's a huge problem, I think, with communication, Sami communication is non-existent … we're supposed to understand each other, both out in the forest and socially, without talking. And … of course that doesn't always work.

Some [people], they keep on and work a whole lifetime and have done it wrong the whole time, I mean, not a single person has told them.

In the end, they have to believe that things will get better if they endure, and if they do not, but give up, they know this will be seen as a failure, that they did not have “the right interest”:

I think quitting and getting another job, that would be like defeat, not making it as a reindeer herder, “he failed” … I mean, those who are reindeer herders are born into it.

### The impacts and meanings of relations

Support from family and close relatives is very important, more important than the Sami village, which nonetheless shows internal loyalty in relation to other Sami villages even if there are internal conflicts. It is your family you turn to if you have problems, for example, to your father or older male relatives if you have practical problems or have come in conflict with other reindeer herders. Inner ruminations may be vented with a mother or girlfriend, but not usually with other relatives or friends. The family is a kind of system of service you can rely on when you need practical help, and you are supposed to be there, show loyalty and stick together. Within the family you learn the norms and values that you pass on to your own children. One informant expressed it thus:

I can say so much, that … within the family within the Sami community, I think we take care of each other, pretty much, and sure, you want people around you within that circle to maybe do what you do because that generates help [to yourself].

Each group keeps to its own both because of outer pressure from the society they are not a part of, and because of inner, vaguer pressure from the norm system:

It's not that beneficial at all to feel that you can't be a part of the society we live in. Because it's too heavy to go around sometimes among … among regular … among non-Sami … It's too heavy … So we surround ourselves with reindeer herders and Sami … to cope with the … the pressure from society.

Sometimes it is good to have non-Sami friends to speak with about other subjects. But the pressure to stick to one's own group can make others react negatively if you have close relations outside the community: “My last girlfriend … couldn't handle me having so many Swedish friends.”

### Being a young reindeer herder means so many (impossible) dreams and conditions

A common dream among the informants is to live as a reindeer herder without the pressure they experience today. They would like to live as they were raised, with the freedom and pride they feel when they are with the reindeer, and to be in control of their lives and their future. But actual conditions differ greatly from this ideal. Experiencing tremendous problems and worries, together with a feeling of powerlessness, makes them feel it is virtually impossible to attain this dream. They feel they have been given a gift that only the very few receive, to be born and raised in a reindeer herding family, but it is both their privilege and their burden.

## Discussion

### Limitations

The study attempts to shed some light on experiences of what it is to be a young male reindeer herder. We acknowledge some methodological limitations. The number of reindeer herders interviewed was small, and the request to participate in the study was made through personal contacts, which may have induced selection bias. We specifically asked for young men who were willing to share their experiences and who had the time to meet us, and this might have narrowed the group. Credibility and trustworthiness for this study are supported, however, by participant selection and the description of the research process, the careful outlining of the steps involved in the analysis and by the use of quotations in presentation of the results (23). The matter of interviewer–interviewee relationship was specifically addressed in the research process, in relation to its impact on the quality of the interviews (24). Even though the impact of these relations was consciously discussed in the research group, there might still be undetected influences depending on the background of the interviewer. One interviewer was “non-Sami,” and one with the specific position of “having partly left reindeer-herding to study on the University,” the latter in a position that the participants discussed as a non-alternative for them. There might also have been a lacking of nuances in the interviews with participants who also speak Sami. The participants did not express this and it was not perceived by the interviewers. Nevertheless, being Sami speaking or not is a sensitive subject, since the language is a part of the cultural identity. Therefore we cannot be certain in our understanding of the impact of the chosen language. Even with the limitations mentioned above, we feel our study contributes to furthering the research on Swedish Sami reindeer herders.

## Discussion of results

The study reveals several important findings regarding young Sami men's experiences of being reindeer herders and these experiences relation to the health situation for the group. The overarching theme that emerged was “being a young reindeer herder means so many (impossible) dreams and conditions.” The young men in this study found themselves caught between the feeling of being one of a chosen few, with the chance to carry on the tradition of reindeer herding, and a feeling of powerlessness against the many obstacles and unjust circumstances that make reindeer herding almost impossible. These circumstances are, for example, unjust legislations regarding financial conditions, predators, intrusion from mining companies and conflicts on several levels. In both this study and others (2) there is a significant and substantial difference between being a reindeer herder and not being one. There is also a circular argument of “interest,” resulting in the fact that someone who gives up (who had the opportunity to be a reindeer herder but quit) is considered “defective” in some way. The choice as to whether to be a reindeer herder or not is not presented as depending on the surrounding circumstances. Instead, the main factor is if you have the right “interest,” and if you do, you continue. And as every reindeer herder is brought up in a reindeer herding family, they should have learned to have this “right interest.”

The line between being and not being a reindeer herder should be understood in the light of history and acculturation. When the Swedish Government realized the consequences of past treatment of the Swedish Sami, they decided to “rescue” reindeer herding as an expression of Sami culture. Through legislation it was indirectly decided which form of reindeer herding was appropriate and that reindeer herders should not have other occupations, for example, by putting up obstacles to the original hunting, fishing and reindeer-keeping *(ärjemarkshushållning)* way of life carried out by Sami for years. Those who chose to be reindeer herders were not even allowed to live in ordinary houses so as not to acclimatize to a Swedish lifestyle for which they were not “made.” Sami who for various reasons were not able to subsist on reindeer herding were forced to become “Swedes” in order to be allowed to build houses or enter other occupations than reindeer herding, thereby losing their right to their own territories, *lappskatteland* (formerly viewed and taxed on a par with landowning) (11). As the Swedish Government's policy and laws were aimed only at reindeer herding Sami, the reindeer herders were separated in their own group and the other Sami were assimilated into the majority culture.

The young men in this study describe how all other Sami cultural symbols, such as language and crafts, are now secondary, implying a situation where reindeer herders have adopted the leadership's (Swedish Government's) core belief (25) that a Sami, by definition, is a reindeer herder. This is also revealed in their descriptions of a certain longing for a “traditional” reindeer herding lifestyle, that is being a skilled reindeer-herder that herd and owns very many reindeers and making a living of only reindeer-herding. In reality, this only existed for a few individuals during a short period of time during the golden age of reindeer herding, and thereby is not as “traditional” as is commonly perceived (11). However, this perception has many resemblances with the Swedish Governments misunderstanding of the Sami people during the 19th century. In this context the young men describe a multilevel understanding of the importance of being a reindeer herder: the importance of “living as I want to, in close contact with nature and reindeer”; of not being a failure, for the sake of preserving the family name in the reindeer herding culture; of not disappointing the family; and from the perspective of not being able to return once they have left. This is understood as a free choice, but the choice is made in strong relation to important people and structures, free on the one hand but also more obligating and bound than anything else they know of.

This aspect of “the reindeer is one's first responsibility” also implies that a reindeer herder is a man, without primary responsibility in the area of everyday childcare. There are also supporting arguments regarding economics (at least one spouse needs to bring home regular pay) and physiology (heavy motorized management). In this important, wonderful, strictly Sami-identifying, culture bearing, gender-segregated and obligating position, every experience of threat should be considered serious and requiring of attention. As such, the young reindeer herders' experiences of threats and powerlessness depending on governmental legislations are vast: constant economical pressure, predators dispersing and killing the herd, and exploitation of grazing lands. Other problems are conflicts with landowners, other Sami villages and within one's own village. Both threats and powerlessness are critical issues in the conceptualization of anxiety and depression (26,27), which both are related to problems regarding alcohol abuse and suicide ideation (28,29). Experiences of offensive and prejudiced media descriptions of reindeer herders and the feeling that people outside the reindeer herding community lack understanding makes it hard for young Sami reindeer herders to communicate outside the Sami community, especially when they feel they do not have the support of the Swedish Government – quite the contrary. Therefore they communicate mostly within their own group, which can also be understood from a historical perspective, as the Sami people have traditionally avoided conflicts with governmental representatives to such a degree that the Swedish courts have even used it as an affirmative for legislation regarding the Sami (11). In this study, the young reindeer herders expressed a deep sense of distrust towards the Government. There was a feeling among them that the Government acted as if reindeer herding was a dying culture that existed only through the graces of the Swedish society. This sense of lacking understanding from persons outside the Sami community may seem somewhat contradictory to the experiences of having high status in the Sami community. This complex situation is nevertheless not contradictory, it is rather an important finding in this study.

In relation to avoiding conflicts, a mentality of sacrifice was mentioned by the informants, but, even though they were not happy with this position and did not know how to make the appropriate changes, they hoped there would be changes in the future and that they and their offspring would still be reindeer herding by the time the changes came about.

### Health, culture and work

Born and raised under Sami reindeer-herding circumstances, the men in this study distinctly experience this specifically developed norm culture. One should have the right “interest” and the right norms and values. You should not be weak, and therefore you do not show any possible weakness, which leads to a culture in which each man is expected to solve his own problems and to endure, also described as a typical male culture. Psychological problems are solved in the same way as practical problems, by working harder and “biting the bullet,” while hoping for a brighter future, a coping behaviour that in research has been described in relation to mental health problems and suicide among young men (30–32). This may be interpreted as related to the North Sami term “birget,” meaning “to manage, to get by.” “Birget” differs from “male norm culture” in that matter that it is used for both men and women, for reindeer-herders and “not reindeer-herding Sami,” while the described norms mainly concerns the present group, reindeer-herding men. As “birget” is an old symbolic term, an analysis in the light of “birget” would demand a study more aimed to investigate gender and cultural development.

Experiencing this norm the young men also described its lack in functionality, for instance, when they were not able to realize that a friend was contemplating committing suicide or when people in their surroundings do not understand that they are doing things the wrong way. These norms might also be understood as contributing to future difficulties, for example, how to reach out to reindeer herders with psychiatric services and suicide preventive strategies offered by the main Swedish society, a problem shared by other indigenous populations (17,19,33). The most important buffer against psychological problems was expressed as being the family, and the informants described the great need they felt of support from their closest relatives. Practical and psychological help are divided among different family members, but both are taken care of within the family. One aspect in this context is that the reindeer herders often had little contact with relatives who were not reindeer herders, which sometimes reminded them of what it would be like if they quit the reindeer herding business.

##  Conclusion and implications

This study shows that the group of young reindeer herding Sami men experience difficulties on many levels and that they feel that there is little they can do about their situation but to “bite the bullet” and endure. In a work health context (34–36), this position is associated to stress and stress-related mental health problems. This situation may partly be explained as a result of Swedish legislation that both historically and presently divides and separates reindeer herders from other Sami. This may in turn create a strict division between being a reindeer herder and not being one, a division that is described as an important factor to the young men. Under present circumstances the young male reindeer herders experience a great deal of injustice and lack of understanding from society as well as mistrust against the Government, which is understood as acting against reindeer herding. An important factor is the described typical male culture that tells you not to show weakness but to “bite the bullet,” endure and hope that everything will work out in the future. This could be described in summary as “being a young reindeer herder means so many (impossible) dreams and conditions.” We believe this knowledge can provide a basis for future interventions and contribute to a broader perspective of strategies for a positive development of reindeer herding in general and the mental health of reindeer herders in particular. These strategies should be specifically aimed to enhance collaboration and social support and to lower barriers to help seeking behaviour.
